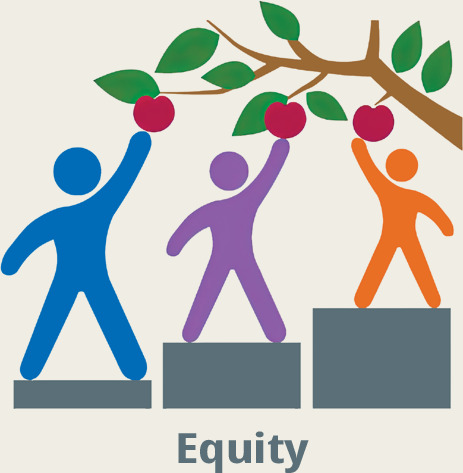# Achieving gender equity in eye health

**Published:** 2025-03-07

**Authors:** Louisa Syrett, Sumrana Yasmin, Jacqueline Ramke

**Affiliations:** 1 Head of Gender Advocacy and Engagement: The Fred Hollows Foundation, UK.; 2 Deputy Technical Director, Eye Health: Sightsavers, Islamabad, Pakistan.; 3 Associate Professor, Global Eye Health: International Centre for Eye Health, LSHTM and School of Optometry and Vision Science, University of Auckland, New Zealand.

**To address the current imbalance in eye health between men and women, we need to do more, and do it better.**

**Figure:**
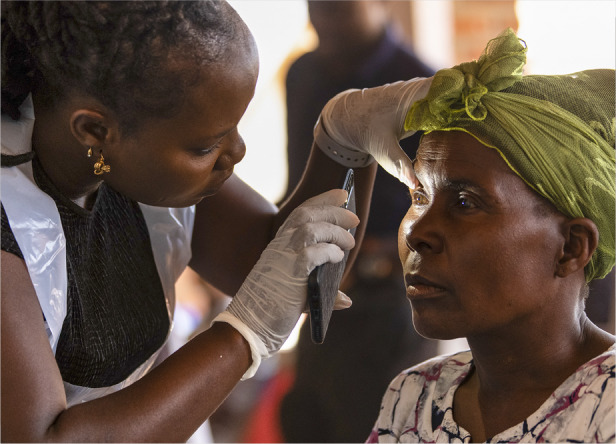
Women make up 70% of the health workforce. malawi

© SIGHTSAVERS/THOKO CHIKONDI CC BY-NC-SA 4.0

We are delighted to present this issue on eye health for women and girls. This is the first time since 2009 that the journal has focused on gender equity. Women and girls – and gender-diverse people – still experience worse access to good quality services, and therefore have a higher prevalence of vision impairment compared to men and boys. While the articles in the issue focus on women and girls as the largest group experiencing gender inequity, we recognise the amplified challenges faced by gender minorities, including transgender and non-binary people.

Women and girls make up 55% of the 1.1 billion people experiencing vision loss. The fact that women tend to live longer than men, and have higher rates of vision impairment due to age-related conditions, does not fully account for this difference in eye health outcomes between genders. The key cause is a lack of equitable access to high quality eye health care, due to both systemic barriers and social and cultural factors.

Eye care services are not reaching enough women. It was estimated in 2022 that effective cataract surgical coverage (eCSC) was 3.5% lower in women than men, while effective refractive error coverage (eREC) was 10.4% lower in women. In some regions this gap is even larger: for example, the eCSC gender gap in the Eastern Mediterranean region is more than twice the global average.

Women's higher prevalence of vision impairment means they disproportionately experience consequences to health, education, and economic stability, leading to reduced quality of life and increased mental health risks, contributing to a cycle of poverty and gender inequity.

The good news is that there are solutions to address gender inequity. A critical first step is to use gender-disaggregated data for advocacy at a policy level, as well as for planning more gender-responsive services.

It is just as vital to listen to women. Their experiences are shaped not only by their gender, but also by factors such as their age, socioeconomic and disability status, and ethnicity; a concept known as intersectionality. Developing targeted strategies to overcome these diverse barriers is only possible through consultation with women and girls representing all of these groups.

Action at a programme management level includes educating health workers on gender biases, engaging men and boys as advocates and bringing eye care closer to communities. Actions at the clinic level includes introducing gender-specific waiting lines and accommodating childcare needs. Read more in this issue, and make use of the excellent guidance available in the IAPB Gender Equity Toolkit: bit.ly/IAPBgender

## Part of the solution

Women make up over 70% of the global health workforce, but only hold 25% of leadership roles, creating a significant gender gap. Women leaders are well placed to understand the unique challenges faced by underserved women and girls, and there is substantial evidence (bit.ly/WDEHfhf) that having more women in leadership leads to better outcomes, innovation, productivity, retention, and financial performance. In health, this means more effective, inclusive, and sustainable health care systems.

We must actively cultivate strong female leadership at all levels of the eye health sector. This includes promoting mentorship and skills-building, and providing equal opportunities for women's advancement. A global monitoring project is the biennial survey implemented by the IAPB Gender Equity Work Group, which highlights best practices and areas for improvement – also available in the IAPB Gender Equity Toolkit (bit.ly/IAPBgender).

Achieving gender equity in eye health not only enhances women's own health and wellbeing, but also provides substantial societal and economic benefits.

*We are grateful to the many contributors to this issue: the authors, who shared valuable experience and learning, the reviewers, who provided constructive comments and advice along the way, and the CEHJ editorial team.*

**IAPB Gender Equity Toolkit**

bit.ly/IAPBgender

**Figure:**
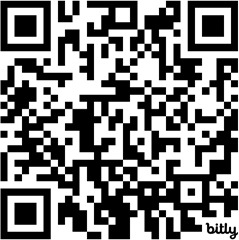

**Equality vs equity**

**Equality** means providing everyone with the same resources and opportunities, aiming for uniform treatment regardless of individual circumstances.

**Figure:**
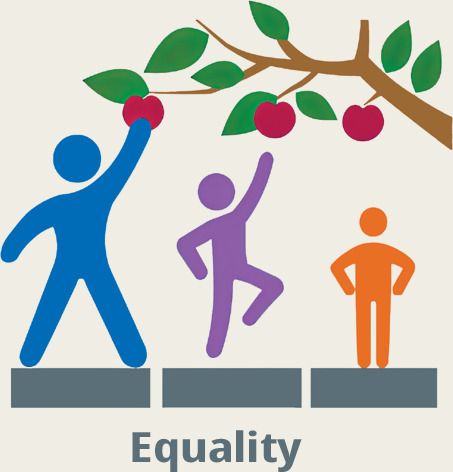

**Equity** recognises that people have different needs and circumstances, and it involves offering varying levels of support to ensure fair outcomes. In essence, equality is about sameness, while equity focuses on fairness and accommodating diverse needs.

**Figure:**